# Choroidal thinning in myopia is associated with axial elongation and severity of myopic maculopathy

**DOI:** 10.1038/s41598-024-68314-w

**Published:** 2024-07-30

**Authors:** Momoka Midorikawa, Kiwako Mori, Hidemasa Torii, Yohei Tomita, Yan Zhang, Kazuo Tsubota, Toshihide Kurihara, Kazuno Negishi

**Affiliations:** 1 Department of Ophthalmology, Keio University School of Medicine, 35 Shinanomachi, Shinjuku-ku, Tokyo 160-8582 Japan; 2 Laboratory of Photobiology, Keio University School of Medicine, 35 Shinanomachi, Shinjuku-ku, Tokyo 160-8582 Japan; 3 Chorioretinal Biology, Keio University School of Medicine, 35 Shinanomachi, Shinjuku-ku, Tokyo 160-8582 Japan; 4 Tsubota Laboratory, Inc., 34 Shinanomachi, Shinjuku-ku, Tokyo 160-0016 Japan

**Keywords:** Choroidal thickness, Myopic maculopathy, Axial elongation, Health care, Medical research

## Abstract

High myopia can lead to pathologic myopia and visual impairment, whereas its causes are unclear. We retrospectively researched high myopia cases from patient records to investigate the association between axial elongation and myopic maculopathy. Sixty-four eyes were examined in patients who visited the department between July 2017 and June 2018, had an axial length of 26 mm or more, underwent fundus photography, and had their axial length measured twice or more. The average axial length was 28.29 ± 1.69 mm (mean ± standard deviation). The average age was 58.3 ± 14.4 years old. Myopic maculopathy was categorized as mild (grades 0 and 1) and severe (grades 2, 3, and 4). The severe group had longer axial lengths than the mild group (*P* < 0.05). Moreover, the severe group exhibited thinner choroidal thickness than the mild group (*P* < 0.05). When subjects were grouped by axial elongation over median value within a year, the elongation group showed thinner central choroidal thickness than the non-elongation group (142.1 ± 91.9 vs. 82.9 ± 69.8, *P* < 0.05). In conclusion, in patients with high myopia, the severity of maculopathy correlated with choroidal thickness and axial length. Thinner choroidal thickness was associated with axial elongation based on the baseline axial length.

## Introduction

Myopia is a condition in which the image is focused anterior to the retina. A more advanced degree of myopia causes transformation of eyeballs, which may lead to visual disturbance called pathologic myopia^1–3^. In the classification of pathologic myopia in the META-PM study (meta analyses of pathologic myopia), pathologic myopia was defined as the eyes having chorioretinal atrophy equal to or more severe than diffuse atrophy. Such eyes may develop visual loss due to various pathological changes in the posterior retina, the peripheral retina, and the optic nerve^4^. Globally, especially in East Asia, the number of myopia patients is increasing and has become a major social problem. It is estimated that by 2050, nearly 50% of the global population will be affected by myopia^5,6^. As the degree of myopia increases, the risk of developing posterior segment disorders such as myopic maculopathy, macular holes, and glaucoma, leading to visual impairment, also rises^7^. However, the mechanisms underlying the development of myopia remain unclear. While it is evident that myopia development is based on axial elongation, the specific factors contributing to this elongation process are still not fully understood.

Myopic maculopathy is a characteristic macular lesion found in the posterior pole of eyes with pathologic myopia. In 2015, the Meta-analysis of Pathologic Myopia (META-PM) Study Group introduced international diagnostic guidelines for pathologic myopia. The classification of myopic maculopathy is based on fundus photography and includes categories such as "no lesion" (Category 0), "tessellated fundus" (Category 1), "diffuse chorioretinal atrophy: D" (Category 2), "patchy chorioretinal atrophy: P" (Category 3), and "macular atrophy" (Category 4). Additionally, lacquer cracks (Lc), myopic choroidal neovascularization (CNV), and Fuchs' spot (Fs) are considered positive lesions^8^. Eyes exhibiting posterior staphyloma or lesions greater than diffuse atrophy are defined as having pathologic myopia^8^. The risk of myopic maculopathy increases exponentially with the severity of myopia^9^. The Tajimi Study in 2006 revealed that myopic maculopathy accompanying pathologic myopia was the leading cause of monocular blindness, accounting for 22.4% of blindness cases^10^.

The choroid, located between the retinal pigment epithelium and the sclera, plays roles in suppressing and absorbing light scatter, providing nutrients to the outer retina, and regulating eye temperature. When the retina detects defocus signals, the choroidal thickness modulates to adjust the focus of the eye to the image^11^. It is known that the choroid becomes thinner with increasing refractive error and axial elongation in myopia^12^. Furthermore, several studies have reported choroidal thickness to be associated with high myopia or myopic maculopathy^13–16^. However, risk factors for the progression of myopic maculopathy have not been clearly identified. To adequately explain the relationship between choroidal thickness and myopic maculopathy, more research is needed.

In this study, we extracted cases of high myopia from the medical records of patients who visited the Keio University Hospital Department of Ophthalmology and investigated the relationship between axial length, in the International Classification of Myopic Maculopathy, and choroidal thickness.

## Results

### Patient characteristics of the entire cohort

Patient characteristics of the cases are presented in Table 1. The total number of cases was 64 eyes, comprising 32 eyes of male patients and 32 eyes of female patients. One eye is used for one case. The mean age was 58.3 ± 14.4 years, with the youngest at 22 years and the oldest at 81 years. The average axial length was 28.29 ± 1.69 mm, and the mean choroidal thickness was 111.1 ± 85.7 µm. The site for measuring choroidal thickness is the central choroidal thickness. The average observation period for axial length was 4.3 ± 2.2 years, with the longest being 7.3 years and the shortest being 0.2 years. All patients were phakia and there were no cases that underwent surgical interventions during the course of observation.

**Table 1:** Patient background of all cases.

Number of cases	64 eyes
Gender	Men 32 eyes, women 32 eyes
Average age	58.3 ± 14.4 years old (maximum 88 years old, minimum 22 years old)
Average eye axial length	28.29 ± 1.69 mm
Average choroidal thickness	111.1 ± 85.7 mm
Axial length observation period	4.3 ± 2.2 years (maximum 7.3 years, minimum 0.2 years)

### The degree of myopic maculopathy is correlated with choroidal thickness and axial length.

**Figure 1:**
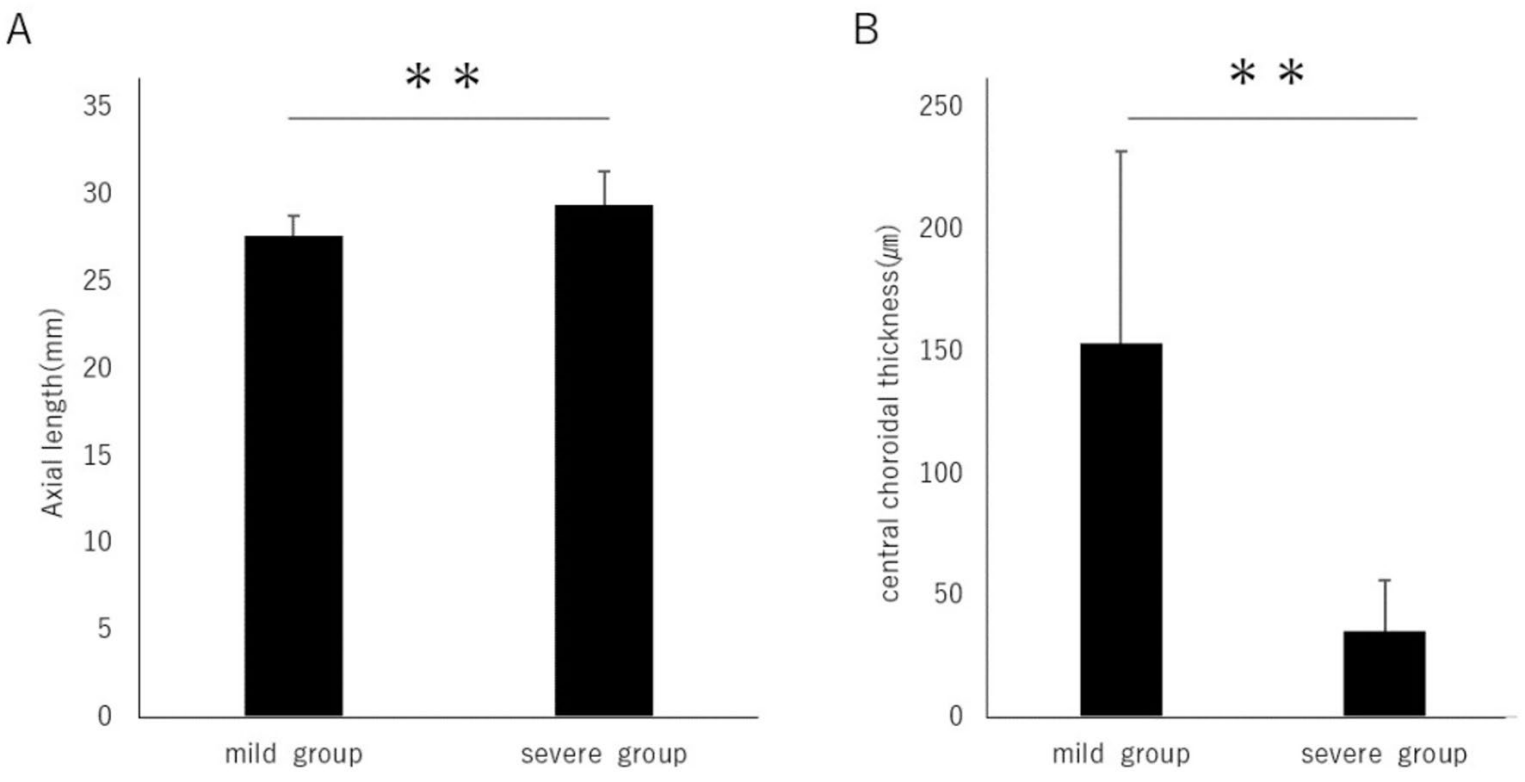
(**A**) When categorizing the international classification of myopic maculopathy as grade 0 and 1 into the "mild group" and grades 2, 3, and 4 into the "severe group," the axial length was significantly longer in the severe group (mild group vs. severe group, 27.65 ± 1.15 vs. 29.42 ± 1.91 mm, *P* < 0.01) (**B**) The choroidal thickness was significantly thinner in the severe group (153.6 ± 78.9 vs. 35.7 ± 20.5 µm, *P* < 0.01). **P < 0.01. Data are shown as mean ± SD.

To investigate the relationship between the degree of myopic maculopathy and choroidal thickness as well as axial length, the myopic maculopathy categories were classified. Categories 0 and 1 were considered as the mild group, while categories 2, 3, and 4 were considered as the severe group (Table 2). The axial length was 27.65 ± 1.15 mm in the mild group and 29.42 ± 1.90 mm in the severe group, and it was significantly longer in the severe group (Fig. 1A). Furthermore, the choroidal thickness was 153.6 ± 78.9 µm in the mild group and 35.7 ± 20.5 µm in the severe group, with a significant thinning observed in the severe group (Fig. 1B).

**Table 2:** Classification of myopic maculopathy.

	Mild group		Severe group		
Category	0	1	2	3	4
Eyes	3	38	15	5	3
%	4.7	59.4	23.4	7.8	4.7

### The association between posterior staphyloma, choroidal thickness, and axial length

**Figure 2:**
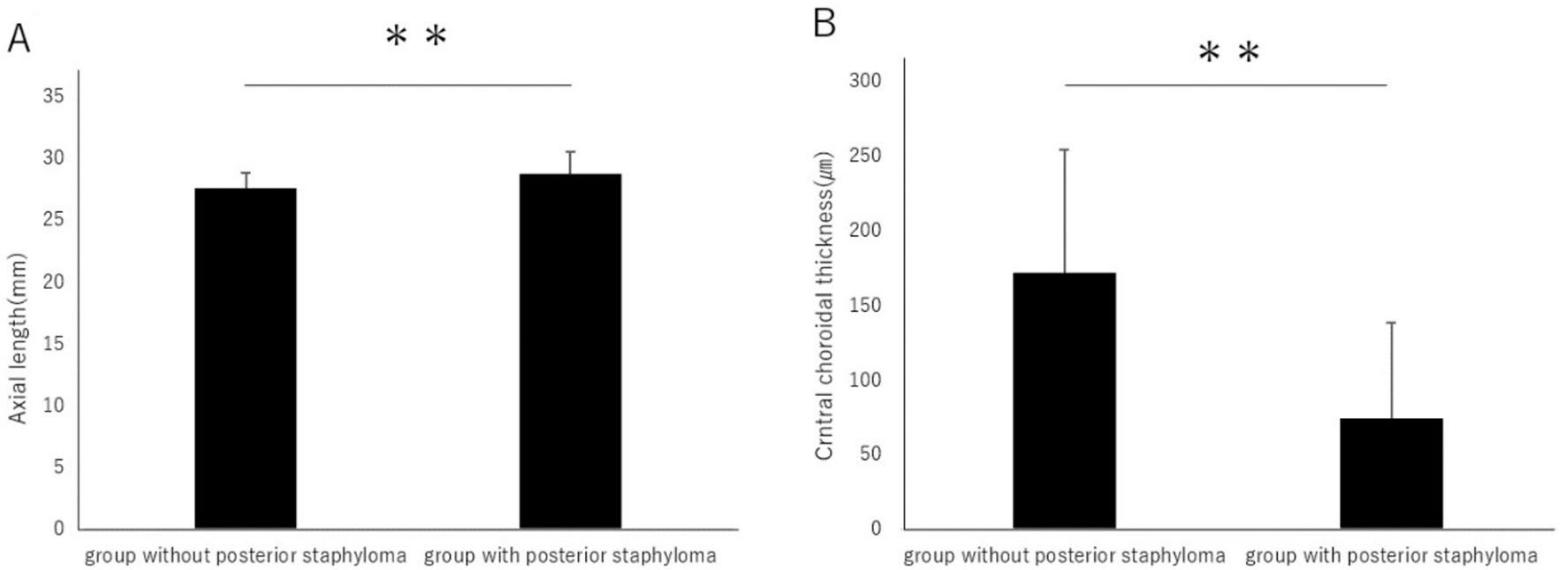
(**A**) The axial length in the group without posterior staphyloma is 27.58 ± 1.23 mm, and in the group with posterior staphyloma, it is 28.77 ± 1.80 mm, showing a significantly longer axial length in the group with posterior staphyloma (*P* < 0.01). (**B**) The choroidal thickness is 171.7 ± 82.9 µm in the group without posterior staphyloma, and 74.4 ± 64.4 µm in the group with posterior staphyloma, indicating that the choroidal thickness is significantly thinner in the group with posterior staphyloma (*P* < 0.01). **P < 0.01. Data are shown as mean ± SD.

A posterior staphyloma is an outpouching of a circumscribed region of the posterior fundus and has been considered a hallmark of pathologic myopia^17^. There are reports that cases with posterior staphyloma have a thinner choroid^18^. In this study, 26 eyes do not have a posterior staphyloma, while 38 eyes do. The axial length in the group without posterior staphyloma is 27.58 ± 1.23 mm, whereas in the group with posterior staphyloma, it is 28.77 ± 1.80 mm, showing a significantly longer axial length in the group with posterior staphyloma (Fig. 2A). Moreover, the choroidal thickness is 171.7 ± 82.9 µm in the group without posterior staphyloma, and 74.4 ± 64.4 µm in the group with posterior staphyloma, indicating that the choroidal thickness is significantly thinner in the group with posterior staphyloma (Fig. 2B).

### The relationship between gender, myopic maculopathy, choroidal thickness, and axial length

**Figure 3:**
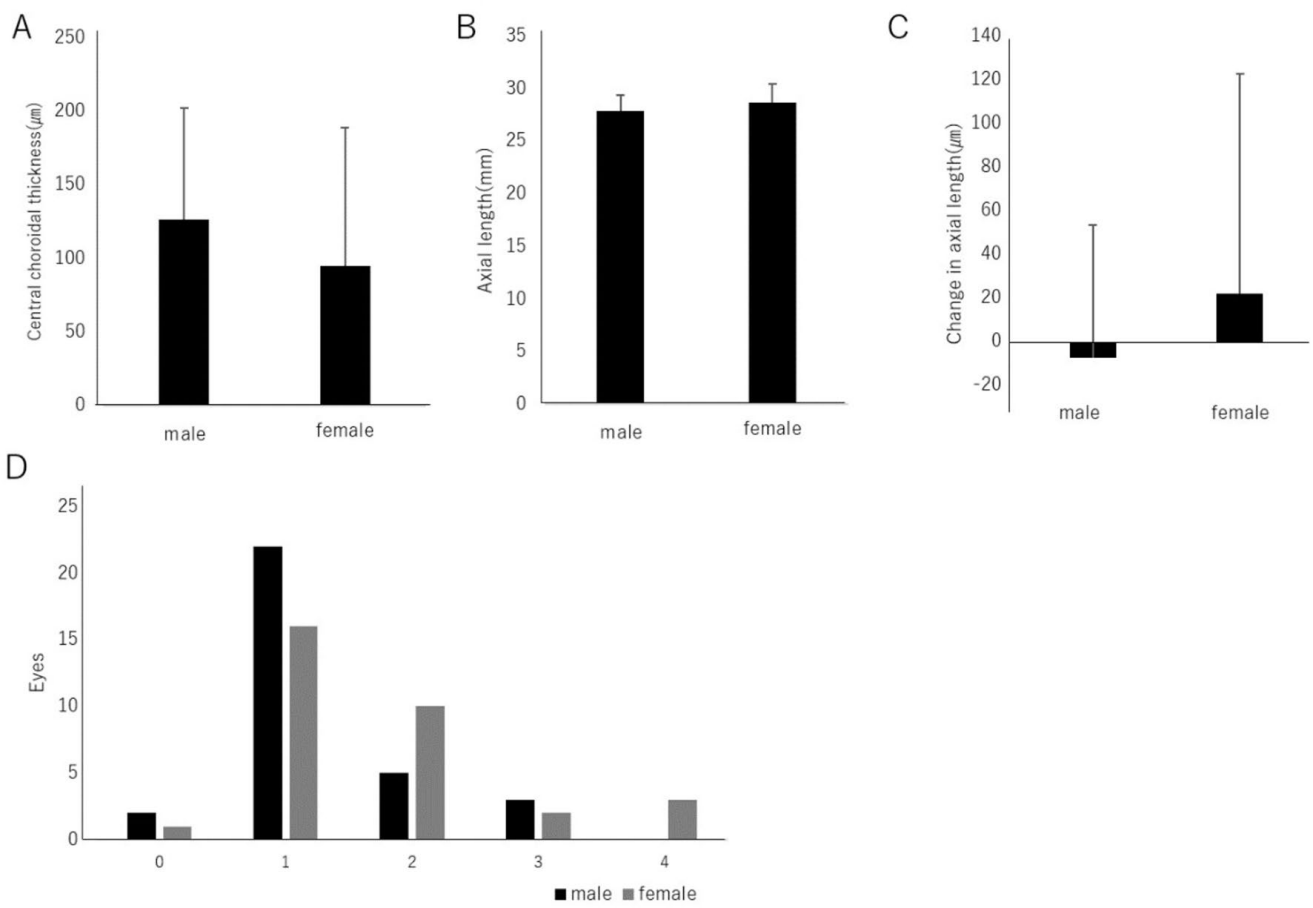
(**A**) The central choroidal thickness in males was 126.4 ± 75.9 µm, and in females, it was 95.2 ± 93.4 µm, with no statistically significant difference observed (*P* = 0.16). (**B**) The axial length was 27.91 ± 1.51 mm in males and 28.66 ± 1.79 mm in females, and there was no statistically significant difference (*P* = 0.07). (**C**) The annual change in axial length was − 7.36 µm in males and 22.06 µm in females, with no statistically significant difference observed (*P* = 0.16). (**D**) There was also no statistically significant difference found in the relationship between the International Classification of Myopic Maculopathy and gender (*P* = 0.07).

Being female is considered a risk factor for the progression of myopia^19^. Therefore, we investigated the relationship between gender and myopic maculopathy, choroidal thickness, and axial length. The central choroidal thickness in males was 126.4 ± 75.9 µm, while in females, it was 95.2 ± 93.4 µm, and no significant difference was observed (Fig. 3A). The axial length was 27.91 ± 1.51 mm in males and 28.66 ± 1.79 mm in females, with no significant difference (Fig. 3B). The annual change in axial length was − 7.35 µm in males and 22.06 µm in females, with no significant difference (Fig. 3C).

No significant difference was found in the relationship between the International Classification of Myopic Maculopathy and gender (Fig. 3D).

### The relationship between axial length elongation, choroidal thickness, and international classification of myopic maculopathy

**Figure 4:**
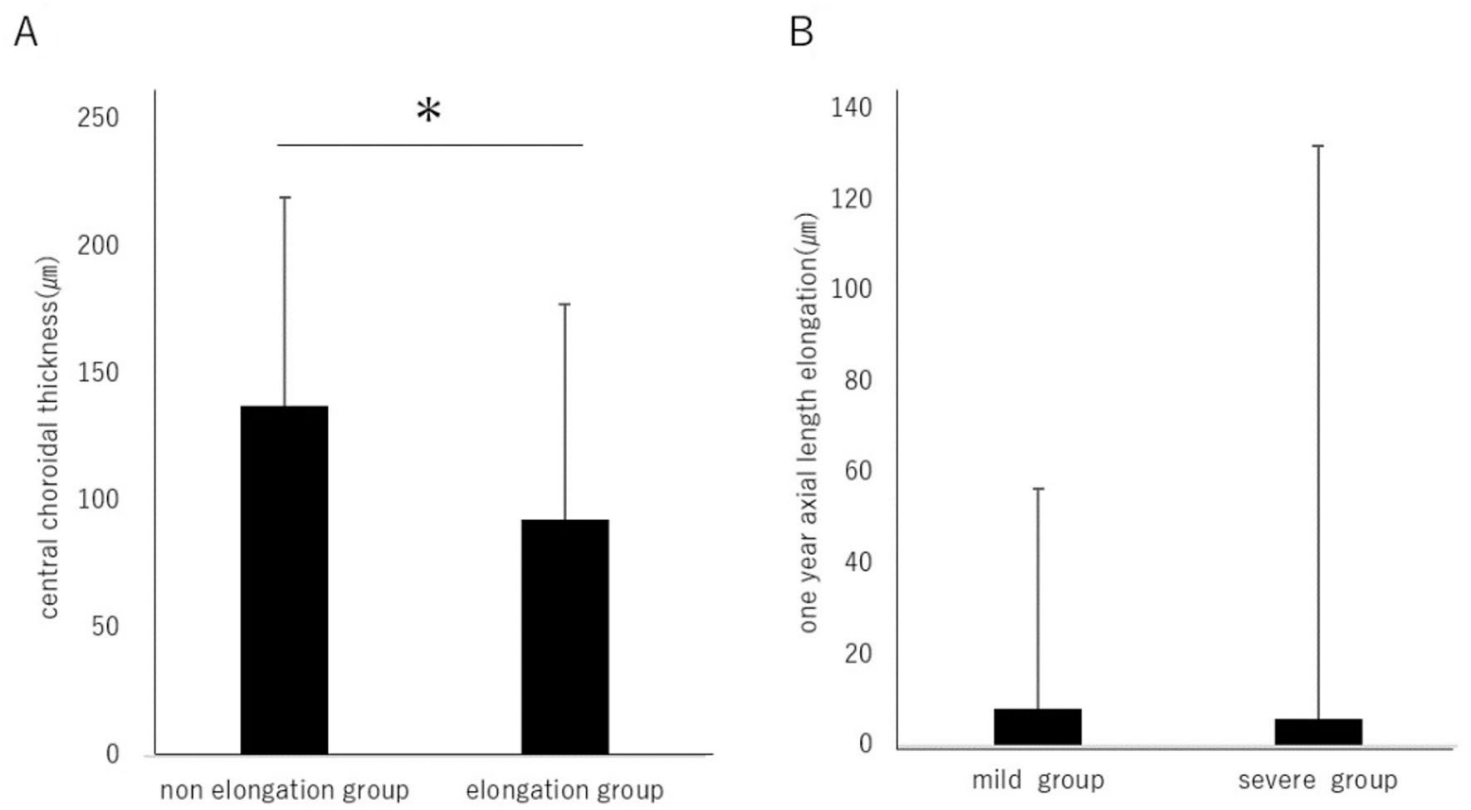
(**A**) The choroidal thickness was 137.2 ± 82.1 µm in the non-elongation group and 92.9 ± 84.5 µm in the elongation group, with a statistically significant thinning observed in the axial length elongation group (*P* < 0.05). (**B**) The axial length elongation over one year was 8.14 ± 48.4 µm in the mild myopic maculopathy group and 5.94 ± 126.0 µm in the severe myopic maculopathy group, with no statistically significant difference observed. (*P* = 0.937) *P < 0.05. Data are shown as mean ± SD.

To examine the relationship between axial length elongation, choroidal thickness, and the International Classification of Myopic Maculopathy, the cohort was divided into groups based on whether the axial length elongated beyond the median change of 5.27 µm over one year. The group with axial length elongation was labeled as the "elongation group," while the rest were categorized as the "non-elongation group." The choroidal thickness was 137.2 ± 82.1 µm in the non-elongation group and 92.9 ± 84.5 µm in the elongation group, with a significant thinning observed in the axial length elongation group (Fig. 4A). Regarding the axial length elongation over 1 year, there was a change of 8.14 ± 48.41 µm in the mild myopic maculopathy group and 5.94 ± 126.01 µm in the severe myopic maculopathy group, without a statistically significant difference (Fig. 4B).

### Factors associated with progression of myopic maculopathy

To investigate factors associated with the progression of myopic maculopathy, logistic regression analysis was conducted. The severity of myopic maculopathy was found to be correlated with choroidal thickness. However, there was no statistically significant association observed between the baseline axial length and gender, as well as between axial length and myopic maculopathy progression (Table 3).

**Table 3:** Factors associated with progression of myopic maculopathy.

	Univariate logistic regression					Multivariable logistic regression					
	OR1	95% CI			*p*-value	OR2		95% CI			*p*-value
Choroidal thickness	0.941	0.908	,	0.974	< 0.001	0.940		0.904	,	0.977	0.002
Axis length	2.063	1.390		3.062	< 0.001	1.598		0.818		3.119	0.107
Female	2.647	0.918	,	7.636	0.072	0.996		0.130	,	7.660	0.997
Axis length elongation	0.730	0.002	,	322.238	0.919	0		0	,	333.054	0.182

OR, odds ratio; 95% CI, 95% confidence interval.

OR1, non-adjusted, OR2, adjusted by choroidal thickness, axial length, gender, and axial elongation.

### Factors associated with axial length elongation

To explore factors associated with axial length elongation, logistic regression analysis was conducted. Axial length elongation was found to be correlated with choroidal thickness and the baseline axial length. However, no statistically significant associations were observed between axial length elongation and gender, as well as between axial length elongation and the International Classification of Myopic Maculopathy (Table 4).

**Table 4:** Factors associated with axial length elongation.

	Univariate logistic regression					Multivariable logistic regression				
	OR1	95% CI			*p*-value	OR2	95% CI			*p*-value
Choroidal thickness	0.991	0.984	,	0.998	0.01	0.987	0.976	,	0.998	0.022
Axial length	1.541	1.096	,	2.166	0.013	1.669	1.025	,	2.716	0.039
Female	2.778	1.010	,	7.643	0.048	3.215	0.910	,	11.354	0.070
Maculopathy severity	1.505	0.539	,	4.207	0.435	0.101	0.012	,	0.834	0.033

OR, odds ratio; 95% CI, 95% confidence interval.

OR1, non-adjusted; OR2, adjusted by choroidal thickness, axial length, gender, and myopic maculopathy.

## Discussion

Myopic maculopathy is a critical complication of high myopia and can potentially lead to vision impairment and blindness. Choroidal thickness has been reported to be associated with the severity of myopic maculopathy^16^. This study confirmed that myopic maculopathy tends to worsen as choroidal thickness decreases. Additionally, it is known that longer axial lengths are associated with thinner choroidal thickness^18^. This study also validated this relationship.

While it was known that myopic eyes tend to have thinner choroids, the underlying mechanism was not fully understood. However, experiments using LDL Receptor Related Protein 2 (LRP2) and Vascular endothelial growth factor (VEGF) gene-altered mice have shown that inducing choroidal thinning leads to axial elongation. Reduced VEGF derived from the retinal pigment epithelium (RPE) causes choriocapillaris underdevelopment, leading to choroidal thinning, which in turn promotes axial elongation and the development of myopia. Adequate levels of VEGF derived from the RPE are necessary for normal eye development to maintain choroidal thickness^20^. Investigating VEGF in relation to myopia could potentially lead to significant advancements in myopia treatment.

In this study, it was found that myopic maculopathy was correlated with choroidal thickness and that thinner choroids were associated with axial elongation. The blood flow in the choroid has been reported to be associated with choroidal thickness^21^. Decreased choriocapillaris diameter and density have been found in a myopia animal model^22^. Additionally, in Jeong et al.'s study, bunazosin hydrochloride (BH), an alpha-1-adrenergic blocker, which selectively inhibits α1-adrenergic receptors in vascular smooth muscle cells and alleviates vasoconstriction, has been shown in animal experiments to suppress choroidal thinning and increase choroidal blood flow^23^. Further research may prove beneficial in maintaining choroidal thickness, preventing axial elongation, and inhibiting the progression of maculopathy by suppressing the reduction of choroidal blood flow.

Advancements in optical coherence tomography (OCT) have greatly contributed to the diagnosis and treatment of retinal and choroidal diseases. This study demonstrated the potential of predicting axial elongation through choroidal thickness measurements. Moreover, given the relationship between choroidal thickness and myopic maculopathy, measuring choroidal thickness might become a predictive factor for the progression of myopic maculopathy. Considering the rapid advancements in myopia treatment, early detection of such abnormalities in highly myopic eyes is crucial. Based on the results of this study, measuring choroidal thickness using OCT in patients with high myopia could become an important indicator for predicting axial elongation and the progression of myopic maculopathy.

This study has several limitations. First, the limited number of cases resulted from the requirement of having at least two measurements of axial length. Second, due to the hospital-based nature of the study, most patients who underwent retinal imaging were expected to have some form of retinal pathology, potentially inflating the prevalence of these conditions. Furthermore, in this study, due to its hospital-based nature, it was assumed that a certain number of glaucoma patients were included, so intraocular pressure was not adopted as an analytical item. This decision was made because it was believed that it might be mistakenly reflected in the multivariate analysis. Third, the measurement time, especially whether AM or PM, for choroidal thickness varies from case to case and therefore is not unified. Fourth, the involvement of outdoor activity time and near-work time is unknown in this study. Fifth, regarding the calculation of axial elongation, per-year elongation is calculated from the degree of elongation for the observational period. Because there is a report that the degree of axial elongation differs in every season, it could be inaccurate that per-year elongation is calculated by multiplying the observed elongation by the number of 1 divided by the corresponding years^24^. Sixth, measuring choroidal thickness was challenging because thin choroids are more common in myopic eyes. Measurement discrepancies were particularly prevalent in cases with thin choroids.

In conclusion, this innovative method of this study revealed that the severity of myopic maculopathy was correlated with choroidal thickness. Furthermore, it was demonstrated that thinner choroids were associated with axial elongation, which may suggest that thinning of the choroid causes axial elongation and as a result affects the severity of myopic maculopathy.

## Methods

### Ethical guidelines

This study was retrospective research conducted in a hospital setting. It adhered to the principles of the Helsinki Declaration, ethical guidelines for medical and health research involving human subjects, and local regulatory requirements. The study was conducted under the approval of all institutional review boards (IRBs) and ethics committees. The study was approved by the Keio University School of Medicine IRB (Approval Number: 20180189). We applied Opt-out method to obtain consent on this study by using a website. The website was approved by the IRB.

### Participants

**Figure 5:**
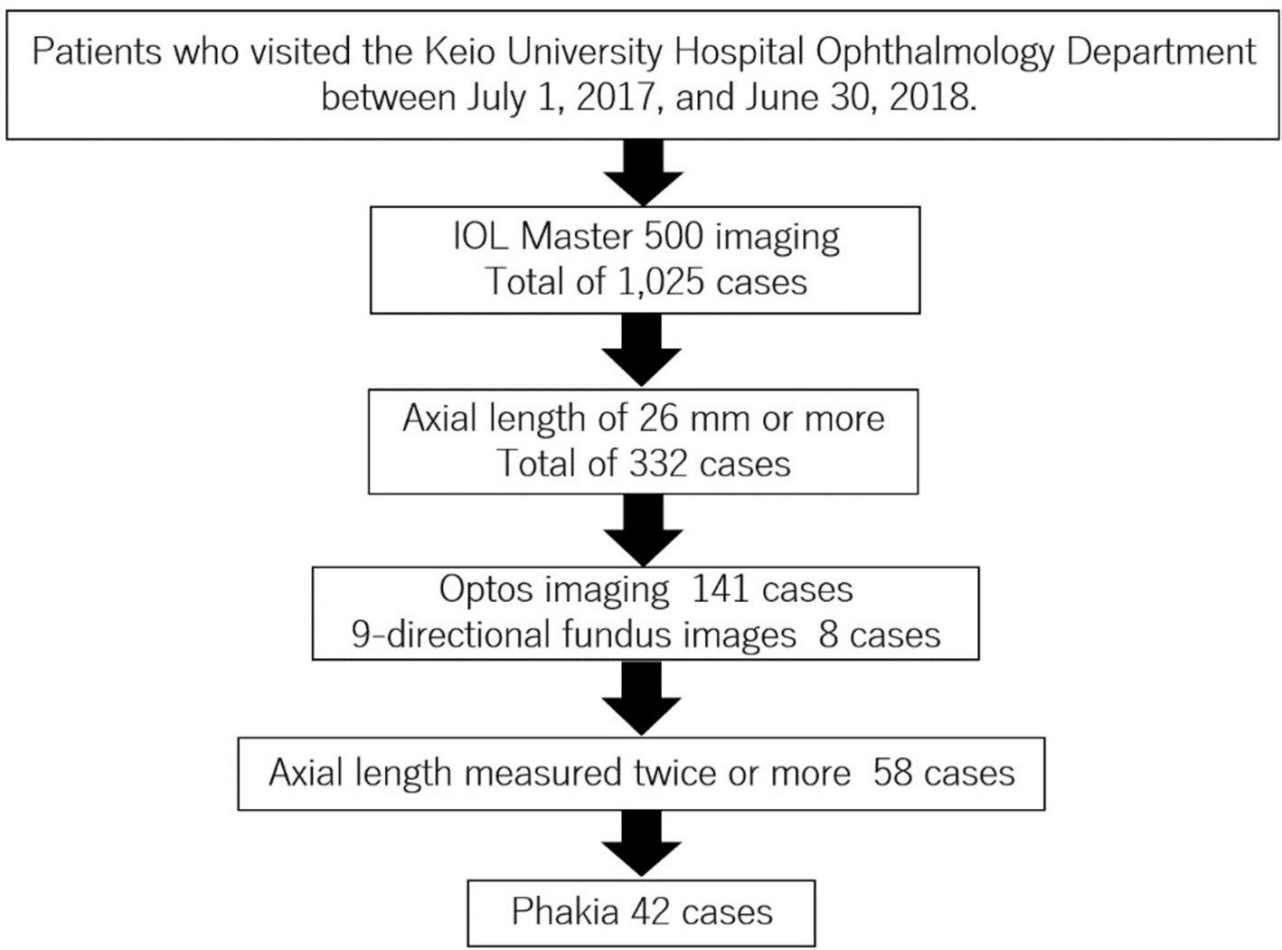
Flow chart of the selection of subjects in this study. We conducted a retrospective study by investigating cases of patients who visited our department between July 2017 and June 2018, had an axial length of 26 mm or more, underwent fundus photography, and had their axial length measured twice or more, based on the medical records.

Data were collected and analyzed from medical records of patients who visited the Keio University Hospital Department of Ophthalmology between July 1, 2017, and June 30, 2018. Unnecessary imaging or laboratory tests were not performed for the purpose of this study. Refraction and axial length are indicators of myopia, but refraction can change due to factors such as cataract surgery history. Therefore, this study utilized axial length as the indicator of myopia instead of refraction. Participants were selected based on the following criteria: axial length measured to be 26 mm or greater (IOLMaster 500, Zeiss, Jena, Germany) through optical biometry, and subjects who underwent ultra-widefield retinal imaging (Optos, Nikon, Japan, or TRC-50DX, Topcon, Japan) capturing 9-directional fundus images, with at least two measurements of axial length taken (Fig. 5). Patients with a history of cataract surgery were excluded.

### Data analysis

**Figure 6:**
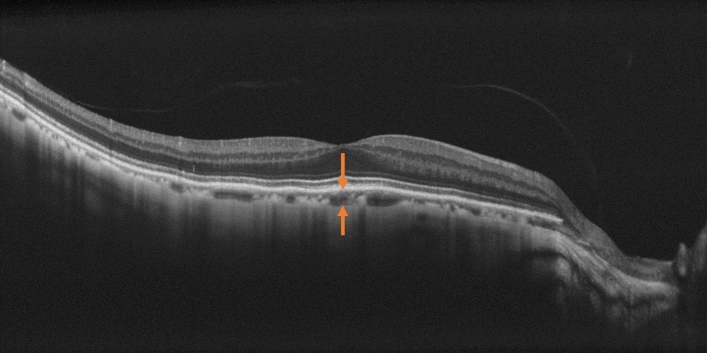
The method for measuring central choroidal thickness. OCT images were captured using the NIDEK RS3000 and the central choroidal thickness was measured from the RPE to the choroid-sclera interface, using built-in scales. This is an OCT image of the right eye of a 66-year-old male with an axial length of 26.70 mm.

Patient information and medical history data were retrieved from medical records. The classification of myopic maculopathy was based on the diagnostic guidelines of the META-PM study. OCT images were captured using the NIDEK RS3000 and the central choroidal thickness was measured from the RPE to the choroid-sclera interface, using built-in scales (Fig. 6). Both measurements and determinations were conducted by two ophthalmologists. The per-year elongation is calculated by multiplying the observed elongation by the number of 1 divided by the corresponding years as a formula below.

$${\text{the\,per}} - {\text{year\,elongation}} = {\text{the\,observed\,elongation }}\times {1}/{\text{the\, observation\,period}}\,\left( {{\text{years}}} \right)$$

### Statistics

Data were presented as mean ± standard deviation. All obtained data were used for statistical analysis. Statistical analysis was performed using SPSS version 27.0 for Windows (IBM, Armonk, NY, USA), employing chi-square tests, t-tests, and logistic regression analysis. Statistical significance was defined as *p* < 0.05. Continuous variables showing parametric distribution were analyzed with Student’s t-test, and variables showing nonparametric distribution were analyzed with the Mann–Whitney U test. Logistic regression was used to estimate odds ratios (ORs) and 95% confidence intervals (CIs).

## Data Availability

All data generated or analysed during this study are included in this published article.
